# Modulation of premotor cortex response to sequence motor learning during escitalopram intake

**DOI:** 10.1177/0271678X20965161

**Published:** 2020-11-04

**Authors:** Eóin N Molloy, Karsten Mueller, Nathalie Beinhölzl, Maria Blöchl, Fabian A Piecha, André Pampel, Christopher J Steele, Ulrike Scharrer, Gergana Zheleva, Ralf Regenthal, Bernhard Sehm, Vadim V Nikulin, Harald E Möller, Arno Villringer, Julia Sacher

**Affiliations:** 1 Emotion Neuroimaging (EGG) Lab, Max Planck Institute for Human Cognitive and Brain Sciences, Leipzig, Germany; 2 Department of Neurology, Max Planck Institute for Human Cognitive and Brain Sciences, Leipzig, Germany; 3 International Max Planck Research School NeuroCom, Max Planck Institute for Human Cognitive and Brain Sciences, Leipzig, Germany; 4 Nuclear Magnetic Resonance Methods & Development Group, Max Planck Institute for Human Cognitive and Brain Sciences, Leipzig, Germany; 5 Department of Psychology, University of Münster, Münster, Germany; 6 Department of Psychology, Concordia University, Montréal, Canada; 7 Division of Clinical Pharmacology, Rudolf-Boehm-Institute of Pharmacology and Toxicology, Leipzig University, Leipzig, Germany; 8 Centre for Cognition and Decision Making, Institute for Cognitive Neuroscience, National Research University Higher School of Economics, Moscow, Russia; 9 Clinic for Cognitive Neurology, Leipzig, Germany; 10 MindBrainBody Institute, Berlin School of Mind and Brain, Charité – Universitätsmedizin Berlin and Humboldt-Universität zu Berlin, Berlin, Germany

**Keywords:** Functional magnetic resonance imaging, neural plasticity, post-stroke motor dysfunction, selective serotonin reuptake inhibitors, sequential motor learning

## Abstract

The contribution of selective serotonin reuptake inhibitors to motor learning by inducing motor cortical plasticity remains controversial given diverse findings from positive preclinical data to negative findings in recent clinical trials. To empirically address this translational disparity, we use functional magnetic resonance imaging in a double-blind, randomized controlled study to assess whether 20 mg escitalopram improves sequence-specific motor performance and modulates cortical motor response in 64 healthy female participants. We found decreased left premotor cortex responses during sequence-specific learning performance comparing single dose and steady escitalopram state. Escitalopram plasma levels negatively correlated with the premotor cortex response. We did not find evidence in support of improved motor performance after a week of escitalopram intake. These findings do not support the conclusion that one week escitalopram intake increases motor performance but could reflect early adaptive plasticity with improved neural processing underlying similar task performance when steady peripheral escitalopram levels are reached.

## Introduction

Motor learning is the improved performance of a motor task following practice^1^ and is modulated by monoaminergic transmission in cortical and subcortical motor networks.^2–4^ Research on this monoaminergic basis of motor learning typically focuses on dopamine signaling in both health^5^,^6^ and disease.^7^ Evidence from rodents^8^ and stroke patients,^9^ however, suggests that serotonin also critically modulates motor behavior. Selective serotonin reuptake inhibitors (SSRIs), commonly prescribed medications for depression and anxiety disorders,^10^ increase extracellular serotonin and successfully treat post-stroke depression.^11^ In the absence of depressive symptoms, several studies have also demonstrated an effect of SSRIs on the recovery of post-stroke motor dysfunction.^12^ Notably, the Fluoxetine for Motor Recovery After Acute Ischemic Stroke^9^ trial showed approximately 50% motor recovery in 57 patients following combined fluoxetine treatment and physiotherapy in a multi-center Randomized Controlled Trial (RCT). These findings were further supported by a meta-analysis of 52 RCTs in 4060 patients, which, however, also acknowledged heterogeneity and methodological shortcomings in a substantial proportion of trials.^13^

Possible mechanisms underlying SSRI modulation of motor performance and learning include anti-inflammatory^14^,^15^ and neurotrophic effects^16^ such as increased neurogenesis,^17^ proliferation,^18^ protein expression enhancement,^19^ upregulation of beta1-adrenergic receptors,^20^ downregulation of γ-aminobutyric acid (GABA)-transmission,^21^,^22^ and hippocampal long-term potentiation.^23^ These findings suggest that SSRIs may increase responsivity to environmental stimuli, possibly via changes in inhibitory and excitatory balance^24^ and reorganization of cortical networks.^25–27^ Studies in humans have provided support for this by demonstrating changes in resting-state functional connectivity induced by a single dose of escitalopram.^28^ Additionally, decreases in resting state alpha-frequency band induced by tryptophan depletion,^29^ which are hypothesized to reflect alterations in the excitatory and inhibitory balance of cortical networks, have been observed in healthy volunteers. Moreover, preliminary functional magnetic resonance imaging (fMRI) evidence has linked decreased functional responses in the motor network with improved motor performance following fluoxetine administration.^30–33^

Recent large-scale RCTs in stroke patients such as the TALOS^34^ and FOCUS trials,^35^ involving over 642 and 3000 patients, respectively, however, do not suggest beneficial effects of SSRIs on functional recovery. Critically, however,^36^ these RCTs were conducted against the backdrop of routinely available rehabilitation and did not combine SSRI administration with a clearly defined motor learning paradigm, nor did they assess functional brain responses to SSRI intake. As a result, no previous study, either in healthy participants or in patients, has successfully leveraged prolonged training on an established motor learning paradigm in combination with SSRI administration and fMRI in an adequately powered sample. Therefore, the hypothesis of whether SSRI administration, specifically in combination with an established motor learning paradigm, induces a beneficial effect on motor learning performance and changes the cortical motor response underlying the learning performance remains to be tested empirically.

The current study utilizes fMRI to address whether one week of SSRI administration in combination with a sequential motor learning task improves sequence-specific motor performance and elicits changes in concurrent cortical motor response during task performance. In a double-blind, randomized controlled pharmaco-fMRI study, we administered 20 mg (to reach 80% serotonin transporter (5-HTT) occupancy)^37^ of escitalopram, the most 5-HTT selective and rapid onset SSRI^38^,^39^ or placebo, to healthy female participants undergoing parallel fMRI assessment and training on a variant of the sequential pinch force task (SPFT).^40^ Given reports of sex differences in (i) motor learning,^33^,^41^,^42^ (ii) fMRI responses during sequential motor control,^43^ (iii) motor responses following SSRI-intake,^33^ and (iv) sex hormone modulation of serotonin transporter density measures^44^,^45^ and escitalopram responsivity,^46^,^47^ we chose a healthy and young (to also control for the effects of pathology^48^,^49^ and age^50^) female sample on oral contraceptives, to avoid variance associated with sex and sex hormones on motor learning performance, fMRI response, serotonin transporter density, and SSRI responsivity. Our a priori hypotheses were (1) that one week of escitalopram intake would improve sequential motor performance relative to placebo, as assessed by performance in a temporal lag condition on the SPFT, calculated as the time difference between a computer-controlled visual stimulus and participant control of a pinch-force device. (2) By specifying this sequence learning condition in an fMRI contrast (hereafter referred to as the learning contrast, i.e. the difference of functional brain responses between two experimental conditions comprising two levels of task difficulty), we also hypothesized escitalopram-induced changes in fMRI response in core components of the motor network during task performance.

## Materials and methods

### Participants and eligibility

Eligible individuals were right-handed, aged 18–35 years, with a body mass index (BMI) 18.5–25 kg/m^2^, without history of neurological or psychiatric illness, and female on oral contraceptives for a minimum of three months. Exclusion criteria were medication use, contraindications for MRI, tobacco use, alcohol abuse, positive drug or pregnancy tests, professional musicianship and athleticism, video game use for more than two hours, on average, per week, and abnormal QT times in electrocardiogram screenings. In total, 88 participants were screened with 71 enrolled. Analyses included 64 volunteers for the behavioral analysis as 6 (escitalopram = 4) chose to discontinue participation and *n* = 1 (placebo) was excluded due to a pre-analytical error in plasma sample acquisition. Sixty volunteers were included in functional imaging analysis as four were excluded due to MRI data quality concerns (two escitalopram) due to head movement. One volunteer from the placebo group was excluded due to an artefact in an anatomical sequence and one participant from the escitalopram group was excluded due to an artefact detected during acquisition of the functional sequence (Supplementary Figure 1).

### Study design and procedure

**Figure 1.**
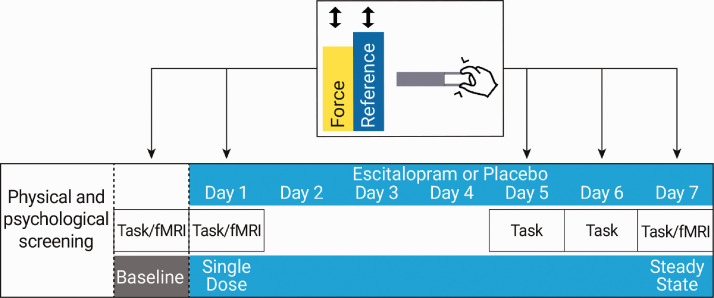
Study design and task: following baseline, escitalopram, or placebo administration took place for seven consecutive days. Post-baseline, motor training took place at single dose (first day), days 5 and 6, and at steady state (day 7). Motor training on days 5 and 6 was completed outside the scanner. fMRI data were acquired at baseline, single dose, and steady state. fMRI: functional magnetic resonance imaging. Notes: Task: sequential pinch force task; Force: the yellow bar controlled by the participants, the rise and fall of which was required to match the rise and fall of the blue (reference bar, i.e. the bar controlled by a computer).

The Ethics committee of the Faculty of Medicine, Leipzig University, approved all procedures (approval number 390/16-ek), as governed by the Declaration of Helsinki of 2013, and the study was pre-registered at clinicaltrials.gov (ID: NCT03162185). All participants provided written informed consent. Participants were randomized to receive either 20 mg of escitalopram or placebo (mannitol/aerosol) orally for seven days. Randomization was performed by the Central Pharmacy of Leipzig University with equal condition allocation. Sequential motor training was conducted five times (baseline, on day 1 of escitalopram administration (single dose), days 5 and 6 of drug administration, and after 7 days administration—steady state) (Figure 1). fMRI data and serum mature brain-derived neurotrophic factor (mBDNF) samples were acquired at baseline, single dose, and steady state. Electrocardiogram recordings were conducted at single dose, day 4, and steady state to monitor potential changes in QT intervals. Adverse reactions to escitalopram were recorded using the antidepressant side-effects checklist (ASEC).^51^ All participants remained under medical supervision during the experiment. Concentrations of escitalopram in plasma were assessed chromatographically using a quality control sample. Deviation of the measured escitalopram concentration of the sample was tested for an acceptance interval of ±15%. All behavioral and fMRI assessments took place three hours after escitalopram or placebo intake to allow for escitalopram to reach maximum levels in serum.^52^

### Sequential pinch force task

We assessed sequence motor learning using a variant of the SPFT, with Presentation (v16.5) running on WindowsXP. Baseline, single dose, and steady-state measurements took place during fMRI, while days 5 and 6 were conducted outside the scanner on an identical separate device. Task completion involved controlling the rise and fall of a yellow bar (force) via the participant’s thumb and index finger (attenuated to individual strength) while attempting to match the speed of a moving computer-controlled blue reference bar (Figure 1). We measured performance in two conditions: (1) a control condition, where the reference bar moves sinusoidally and (2) a sequence-specific learning condition, in which the reference moves in a sequential pattern that remains stable across sessions. A rest condition punctuated training to avoid fatigue. Each session consisted of five blocks with three trials per block and cycled through simple, rest, and learning. Participants received no feedback regarding performance. To assess performance, we calculated the time difference (lag) in milliseconds between the reference and force bar during the learning trials.

### Demographic data and statistical analysis

Independent samples *t*-tests using the R statistical programming language^53^ tested for potential group differences in age, BMI, downregulated hormonal profile, and on total ASEC scores at single dose and steady state. A power analysis conducted using G*Power^54^ (v.3.1.9.4) assuming statistical power of 95% to detect a significant effect of escitalopram on sequence motor learning (i.e. *learning rate over five behavioral assessments compared to placebo)* with a small effect size and an α-level of <0.05 suggested a minimum sample size of 56, with 28 participants per group. To account for potential drop-outs, we aimed to include 60 participants in total.

### Behavioral data preprocessing

All SPFT data were preprocessed using in-house MATLAB scripts. Quality control used an outlier labeling approach^55^ implemented in Python (v2.7.15) in which trial, condition, group, and outcome-specific interquartile ranges were multiplied by a factor of 1.5 to compute upper and lower bound thresholds.

### Behavioral data analysis

All behavioral data analysis were conducted using R. Normality of data distribution was assessed via visual inspection of Q–Q plots and via a Kolmogorov–Smirnov and Shapiro–Wilk test using the factors of group and time, with the Statistical Package for the Social Sciences (version 24).

1. Independent samples *t*-tests assessed baseline group differences using the “t.test” function to assess efficacy of randomization.
2. Comparisons between groups over time employed an omnibus linear random-intercept mixed effect modeling approach using the “lmer” function, within the “lme4” package in R (independent factors: *group, time*, dependent variable: *lag*). Contributions of each fixed effects were assessed with a likelihood ratio test for improvement of model fit.
3. Post-hoc independent samples *t*-tests were conducted on mean single dose and steady state scores to assess potential group differences at each critical time point of escitalopram administration. Additionally, the delta (difference between mean performance at steady state compared to baseline) was compared between groups for each outcome via independent samples *t*-tests. Bayes Factor *t*-tests using the “ttestBF” function in the “BayesFactor” package assessed the likelihood of the null hypothesis for all independent sample analyses.
4. Pearson’s correlation analyses assessed potential associations between total ASEC scores and mean lag performance at both single dose and steady state.

### fMRI data acquisition

fMRI data were acquired with gradient-echo echo-planar imaging on a 3-Tesla MAGNETOM Verio scanner (Siemens, Erlangen, Germany, 32-channel head-coil, flip angle 90°, TR = 2000 ms, TE = 30 ms, field of view = 192 × 192 mm^2^, 30 slices, 64 × 64 matrix, 3 × 3 × 3 mm^3^ nominal resolution, 495 volumes, aligned –15° along the anterior to posterior commissure, ∼16 min). A whole-brain three dimensional T1-weighted Magnetization Prepared Rapid Gradient-echo was also acquired at each time point for co-registration^56^ with inversion time, TI = 900 ms, TR = 2300 ms, TE = 2.98 ms, 1 × 1 × 1 mm^3^ nominal isotropic resolution, ∼9 min.^57^

### fMRI data analysis—Preprocessing and first-level analysis

Data pre-processing was conducted using SPM12 (v12.7219). Data were realigned, unwarped, normalized to Montréal Neurological Institute space, and smoothed with a Gaussian kernel (8 mm at full-width-half-maximum). First-level analysis was performed for baseline, single dose, and steady state separately using a general linear model (GLM) including all three experimental conditions: learning, simple, and rest. In addition, each analysis contained head-movement parameters obtained during preprocessing and motion correction. Following parameter estimation, we generated contrast images specific to sequence learning by specifying the learning contrast (i.e. the difference between the learning and simple conditions).

### fMRI data analysis—Group-level analysis

Using contrast images obtained at the first level, whole-brain second-level analyses were performed in a stepwise fashion with SPM12 in MATLAB (v9.7). Results were considered statistically significant at a whole-brain cluster-defining threshold of *p* < 0.001 corrected at *p* < 0.05 using family-wise error (FWE) for multiple comparisons at the whole-brain cluster-level, following which we applied appropriate Bonferroni corrections for the number of contrasts specified.

1. Following first-level analyses, we investigated the learning contrast (i.e. the difference between the learning and the simple condition) for each group at baseline, single dose, and steady state with a one-sample t-test for each group and timepoint independently. Results were Bonferroni corrected for the number of tests performed (*n* = 6).
2. To assess randomization efficacy, we then conducted an independent-samples *t*-test to assess potential differences in the learning contrast between groups at baseline.
3. In order to identify time points of interest within the escitalopram group, we investigated changes in the whole-brain learning contrast across each time point using a one-way repeated measures ANOVA (escitalopram group at three time points—baseline, single dose, and steady state). Here, we assessed all combinations of paired comparisons using Bonferroni correction for the number of tests performed (*n* = 6). Only time points yielding a significant time effect at this corrected threshold were retained for comparison to placebo.
4. To investigate group differences in the whole-brain learning contrast with respect to results obtained in analyses (3), we specified a flexible factorial model using factors *group* and *time* in which we tested for a *group* by *time* interaction. Subsequent post-hoc paired tests were also performed within the same flexible factorial design including Bonferroni correction for the number of possible tests (*n* = 8).
  4(a). To ensure that results obtained from our whole-brain flexible factorial analysis (4) were not confounded by intra-subject variance in behavioral motor learning performance, we repeated this analysis with an additional regressor. Here, two behavioral measures for each participant (one for each timepoint) were entered as nuisance covariates in the GLM. For these behavioral measures, we used the behavioral sequence-specific learning measure “lag learning-simple score” (LLSS), as calculated for each participant by subtracting the mean simple condition scores from the mean learning condition lag scores.^58^
  4(b). In line with our behavioral analysis, we applied a Bayesian model estimation to investigate the probability of the alternative hypothesis for our interaction contrast. Here, we specified an additional Bayesian model estimation to our flexible factorial design (4) with a medium effect size (equivalent to Cohen’s d = 0.5) and log odds threshold of three, analogous to strong evidence for the alternative hypothesis.^59^
5. Finally, we tested for correlations within the escitalopram group between (i) motor learning performance and changes in the whole-brain learning contrast and (ii) escitalopram plasma kinetics and changes the in whole-brain learning contrast. We used the LLSS and escitalopram plasma levels, respectively, as a variable of interest within two separate flexible factorial designs. Here, each model was generated using the factors *subject* and *time*, and the variable of interest (i.e. *LLSS* or *plasma escitalopram levels*).^58^ Finally, we also investigated potential group differences in patterns of brain-behavior correlations by testing an interaction term between the factors *group* and *LLSS*. Results were Bonferroni corrected for the number of correlational analyses performed (*n* = 3).

### Analysis of serum mature BDNF levels

A one-way repeated measures ANOVA was implemented in R using the “Anova” function to assess changes in serum mBDNF levels across time. Paired samples *t*-tests in both the escitalopram and placebo groups compared baseline to steady state within each group, separately.

## Results

### Demographics

No differences were observed between groups in any baseline screening measures. Escitalopram levels were within the expected range^52^ (Table 1).

**Table 1.** Demographic overview.

	*Escitalopram (n = 31)*	*Placebo (n = 33)*	*t-Values*	*p-Values*
Age (years)	23.90 ± 2.95	22.57 ± 3.72	–0.3	0.74
BMI (kg/m^2^)	21.91 ± 1.65	21.33 ± 1.66	–1.3	0.19
Lutropin (u/l)	2.27 ± 2.78	1.41 ± 1.96	–1.2	0.20
Follitropin (u/l)	3.19 ± 2.89	2.07 ± 3.23	–1.3	0.19
ASEC single dose	3.45 ± 3.32	0.87 ± 1.34	3.9	≤0.001
ASEC steady state	0.74 ± 1.73	0.54 ± 1.06	–0.7	0.48
Escit. single dose (ng/ml)	19.96 ± 5.13	–	–	–
Escit. steady state (ng/ml)	45.48 ± 10.96	–	–	–

Note: group demographic overview and mean single dose and steady-state escitalopram plasma concentrations. Group values refer to mean ± standard deviation.

Escit.: escitalopram; ASEC: antidepressant side effect checklist-score; kg/m^2^: kilogram force per square meter; u/l: units per liter; ng/ml: nanograms/milliliters; BMI: body mass index.

### Sequence-specific motor learning

Using the Kolmogorov–Smirnov and the Shapiro–Wilk test, we did not observe a significant result for motor training sessions one to four. However, for session 5, we observed a significant result (*p* = 0.003 and *p* = 0.001 for each test, respectively). We did not find any significant differences between the escitalopram and placebo groups on behavioral measures of sequence-specific motor learning:

1. Group comparisons of mean performance at baseline did not show any significant group differences in sequence-specific motor learning behavior (*t* = –0.25, *p* = 0.80).
2. For group comparisons over time, a mixed effects model, including a fixed effect of *time*, fit the data significantly better than a random-intercept only model, reflecting a decrease in lag scores (Table 2). The fixed effect of *group* and the interaction of *time* and *group* did not show a significant improvement in fit, demonstrating that, while both groups improved in sequence-specific motor performance over time, they did so comparably (Figure 2).

**Table 2.** Comparisons of nested linear mixed effects models and post-hoc testing for sequence-specific lag scores.

*Mixed effects modeling*	*Fixed Effects*	*Random effects*	*LRT*		*Marginal R2*
			*χ^2^ (df)*	*p-Value*	
Intercept	–	Subject	–	–	0
Time	Time	Subject	3301.3 (24)	≤ 0.001^a^	0.2917
Group	group + time	Subject	0.0181 (1)	0.8931	0.2918
Interaction	group × time	Subject	25.722 (24)	0.3674	0.2934
Post-hoc testing	Escitalopram (M±SD)	Placebo(M ± SD)	*t*-value (df)	*p*-Values	Cohen’s d/BF_01_
Single dose	99.8 ± 57.9	95.1 ± 67.8	–0.3 (62)	0.76	–0.07/0.26
Steady state	58.1 ± 36.1	63.8 ± 44.1	0.56 (62)	0.57	0.14/0.29
Delta scores	–107.1 ± 56.6	–96.7 ± 59.2	0.71 (62)	0.47	0.18/0.31

Notes: Model comparisons for computing the omnibus tests for *group* and *time* as well as their interaction effect for both outcome measures. χ^2^ and respective *p*-values were computed from a likelihood ratio test between nested models with results of independent samples *t*-tests and corresponding Bayes Factors on mean single dose, steady state, and absolute rate of improvement scores (deltas).

BF_01_: Bayes Factor indicating the likelihood of the alternative hypothesis compared to the null hypothesis; M ± SD: means *±* standard deviation; LRT: likelihood ratio test; df: degrees of freedom; χ^2^: Chi-square.

^a^Significant improvement in model fit.

**Figure 2.**
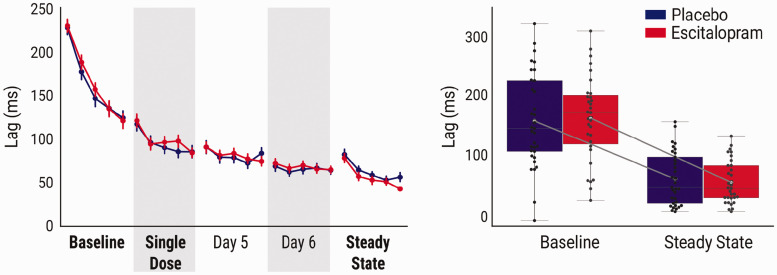
Sequential motor learning. Left: significant improvements in lag scores over five days of sequential motor training across both escitalopram and placebo. However, despite a significant learning effect, we observed no significant group differences in performance, nor did we observe an interaction effect. Right: comparison of the rate of change between baseline and steady state yield no significant group differences. Bold fonts indicate training completed in the scanner.
3. Post-hoc two-sample *t*-tests did not show a significant group difference in mean performance at either single dose or steady state. Comparisons of the delta scores from baseline to steady state did not show any significant differences between groups. Bayes Factor analysis of group comparisons at single dose and steady, as well as the delta, yields moderate evidence in support of the null hypothesis (Table 2).
4. Additionally, correlation analyses did not show an association between total ASEC scores with mean behavioral lag scores at either single dose (*r* = –0.03, *p* = 0.8,) or at steady state (*r* = 0.11 *p* = 0.37), respectively.

### Whole-brain functional MRI responses during sequence-specific motor learning

1. One-sample t-tests across the learning contrast images in each group at each time point show bilateral activation in both the escitalopram and placebo groups at each of baseline, single dose, and steady state (Figure 3).

**Figure 3.**
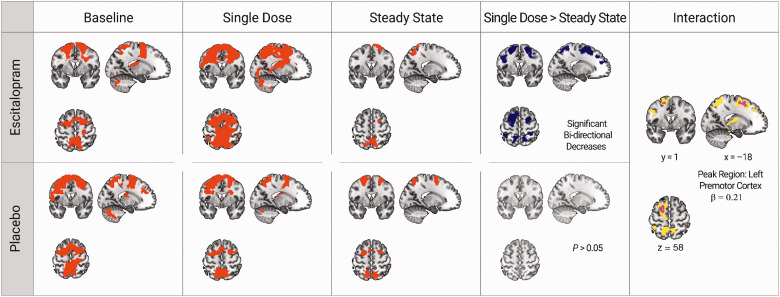
Escitalopram-induced decreases in whole-brain cortical motor responses during sequential motor learning: Orthogonal brain slices showing group-dependent changes in the learning contrast over time. Mean functional group response (red) of the escitalopram group (top) and placebo (bottom) at each baseline, single dose, and steady-state measurements, as computed by a series of one-sample *t*-tests in SPM12. Single Dose > Steady State: brain regions in the escitalopram group with significant decreases in the learning contrast (blue) between single dose and steady state (top row) show decreases in bilateral premotor and temporal–parietal regions (Table 3). Comparisons between single dose with steady state in the placebo group do not yield any significant changes across time (bottom). Interaction: comparisons of groups over time reveal decreases in the whole-brain learning contrast in the left premotor cortex of the escitalopram group that are not observed in placebo (violet). Consideration of behavioral performance as a variable of interest shows brain regions where changes in the learning contrast positively correlate with improvement in motor performance, also with a peak in the left premotor cortex (overlaid in yellow). All results are shown for sequence-specific learning with *p* < 0.05 family-wise error (FWE) correction at a cluster forming threshold of *p* < 0.001. All orthogonal planes presented are the same. β = beta value at global maximum coordinate. See supplementary Table 3 for an overview of significant brain regions corresponding to correlation analyses.
2. We did not observe any group differences in the learning contrast fMRI responses at baseline.
3. Within the escitalopram group, we found a significant decrease of the whole-brain learning contrast in bilateral motor regions when comparing single dose with steady state. We did not observe any significant increases in the learning contrast at any time point.
4. Comparisons of groups over time reveal decreases in the whole-brain learning contrast in the left premotor cortex of the escitalopram group between single dose and steady state that are not observed in placebo (Figure 3, interaction panel, violet overlay). Post-hoc whole-brain results show a significant decrease in bilateral cortical motor regions in the escitalopram group (Figure 3, escitalopram panel, blue), but not in the placebo group.
  4(a). A whole-brain sensitivity analysis controlling for intra-subject variance in task performance replicates this result from analysis (4), showing a significant group by time interaction with a decrease in the learning contrast from single dose to steady state in the escitalopram group in the left premotor cortex (Table 3).

**Table 3.** Escitalopram-induced motor network changes in the learning contrast during sequence motor learning.

*Interaction*	*p(FWE-corr)*	*Cluster Extent*	*t-value*	*z-value*	*MNI (x, y, z)*
Left premotor cortex	0.044	82	4.36	4.04	–21, 11, 50
			4.17	3.88	–15, 8, 59
			4.10	3.82	–18, –4, 59
*Interaction (Sensitivity)*					
Left premotor cortex	0.017	111	4.68	4.29	–21, 11, 50
			4.26	3.95	–15, 8, 59
			4.07	3.80	–27, 5, 56
*Interaction (Bayesian)*	*LogBF Threshold*	*Cluster Extent*	*Cohen’s d*		*MNI (x, y, z)*
Left premotor cortex	3	42	0.5		–18, 11, 56
					–18 –4, 56
*Post-hoc*	*p(FWE-corr)*	*Cluster Extent*	*t-value*	*z-value*	*MNI (x, y, z)*
Left premotor cortex	<0.001	913	6.33	5.50	–18, 11,59
			6.25	5.44	–21, 11, 50
			6.17	5.39	–27, 8, 59
Right premotor cortex	<0.001	233	6.15	5.38	30, –4, 41
			5.10	4.62	24, –4, 65
			4.28	3.97	30, 2, 62
Left superior parietal lobule	<0.001	260	5.82	5.14	–39, –46, 53
			5.33	4.79	–33, –43, 32
			5.13	4.64	–36, –46, 41
Left superior frontal gyrus	<0.001	295	4.87	4.44	–18, 35, 38
			4.53	4.17	–18, 44, 20
			4.31	4.00	–15, 56, 26
Right superior parietal lobule	<0.001	257	4.74	4.34	12, –61, 62
			4.23	3.93	18, –61, 50
			4.06	3.79	33, –40, 59
Left postcentral gyrus	<0.001	215	4.70	4.30	–9, –46, 74
			4.67	4.28	–12, 52, 65
			3.63	3.44	–15, –70, 50
Right middle temporal gyrus	0.002	172	4.67	4.29	42, –58, 11
			3.77	3.55	33, –58, 23
			3.68	3.47	27, –76, 11

Notes: Results of a whole-brain flexible factorial analysis comparing escitalopram and placebo between single dose and steady state with a post-hoc paired comparison (Bonferroni corrected for the number of post-hoc tests performed—α = 0.006) of single dose and steady state within the escitalopram group only. Results significant at *p* < 0.001 (unc) cluster threshold corrected with *p* < 0.05 family-wise error on the cluster level. Also shown are the results from a sensitivity analysis with an additional regressor (sensitivity) and a Bayesian model estimation (Bayesian) of the flexible factorial design.

**Figure 4.**
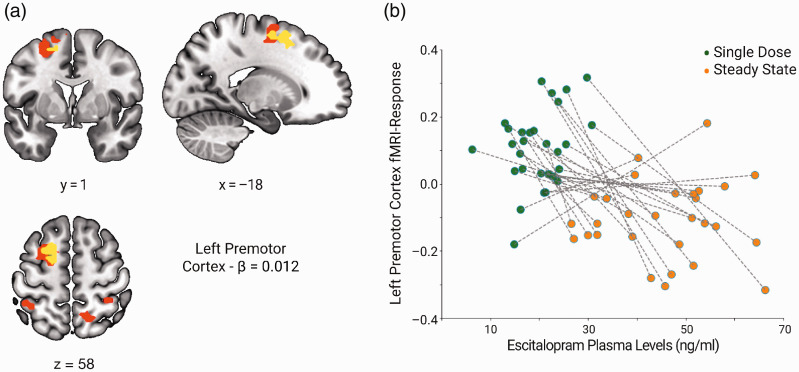
Correlations between escitalopram plasma levels and whole-brain cortical premotor response during sequence-specific learning from single dose to steady state: (a) Escitalopram plasma concentrations negatively correlate with changes in the whole-brain learning contrast in bilateral cortical motor regions, including the premotor cortex (premotor cortex from significant 2 × 2 interaction overlaid in yellow), with a peak in the left supramarginal gyrus. (b) Betas containing parameter estimates for error from the left premotor cortex plotted against escitalopram plasma levels at single dose and steady state, respectively. Results refer to the sequence-specific learning contrast and are shown with *p* < 0.05 family-wise error (FWE) correction at a cluster forming threshold of *p* < 0.001. ng/ml: nanograms/milliliters; β: beta value at premotor MNI coordinates. Note: see supplementary Table 4 for an overview of significant brain regions.

*p*(FWE-corr): *p*-value for whole-brain coordinates surviving family-wise error correction for multiple comparisons; Cluster Extent: number of voxels in cluster; MNI (*x*, *y*, *z*): Montréal Neurological Institute peak coordinates; logBF: Log odd threshold; Post-hoc Escit.: escitalopram group post-hoc paired comparisons.
  4(b). Bayesian model estimation yields a significant cluster in the left premotor cortex. Here, we observed strong^59^ evidence in favor of the alternative hypothesis (Table 3).
5. Correlation analysis between the change in sequence-specific learning performance with the fMRI signal change in the learning contrast from single dose to steady state within the escitalopram group reveals a significant positive correlation in brain regions including the left premotor cortex (Figure 3; yellow overlay, Supplementary Table 3). However, an interaction contrast testing for differences in the pattern of brain-behavior correlations between groups yields no significant group effect. Correlational analysis between escitalopram plasma levels and the learning contrast in the escitalopram group shows a significant negative correlation, with increases in escitalopram plasma concentration associated with decreases in the learning contrast in bilateral regions including the left premotor cortex and supramarginal gyrus (Figure 4, Supplementary Table 4).

### Analysis of mature BDNF levels

Analysis of mBDNF levels from baseline to steady state in both groups combined does not reveal any significant changes over time (F(1, 62) = 2.195, *p* = 0.12). Paired *t*-tests do not indicate significant changes from baseline to steady state in either the escitalopram (*t* = –1.23, *p* = 0.22) or placebo group (*t* = –1.5, *p* = 0.14), respectively.

## Discussion

In this randomized controlled interventional study, we investigated whether administration of 20 mg escitalopram improves motor learning performance and alters functional brain response in the motor network during sequence motor learning. Results show a significant learning effect in sequence-specific motor performance though this rate of improvement does not differ between groups. Additionally, we do not observe any significant group differences at any time point, or in rate of improvement. With a whole-brain fMRI analysis, we find significant escitalopram-induced decreases in the left premotor cortex during sequence-specific learning when comparing single dose and steady state. Moreover, consideration of behavioral performance as a variable of interest during this phase of learning reveals that these changes in the sequence-specific learning contrast positively correlate with improvement in motor performance. Finally, we observe a negative correlation between escitalopram plasma levels and the fMRI response during the sequence-specific learning contrast in brain regions including the left premotor cortex during task-performance, suggesting a parallel development between escitalopram plasma kinetics and the attenuation of cortical motor response to sequence-specific motor learning.

The lack of an effect of SSRI administration on motor learning performance differs from previous findings in healthy vounteers.^31–33^ These studies, however, were neither powered nor pre-registered to test this as an a-priori hypothesis, with 6 healthy volunteers for 5 different behavioral tests and 1 fMRI experiment^31^,^32^ and 19 volunteers for 6 different behavioral assessments.^33^ Additionally, we administered escitalopram and chose a task that may be less cognitively demanding due to repetitive isotonic contractions,^60^ possibly creating earlier ceiling effects in healthy adults. These previous findings could be specific to paroxetine, require tasks with more spatial and coordination-oriented sensorimotor components, or may only become apparent after several weeks of administration. Nevertheless, given that we administered the SSRI with the highest transporter selectivity,^61^ employed a task that reliably measures sequence motor learning,^40^ and tested a sample well-powered to detect robust effect sizes, it is unlikely that this discrepancy is due to the choice of SSRI or motor paradigm alone. Furthermore, our exploratory analysis of mature BDNF levels in plasma did not reveal any significant changes associated with improved motor learning performance in either group. While this is consistent with findings of improved motor performance in healthy volunteers to be unrelated to peripheral BDNF levels,^62^ evidence supportive of an association between motor skill learning and increased BDNF levels have also been reported.^63^ While future studies should assess potential SSRI modulation of motor learning with additional paradigms, our results do not support a beneficial effect of SSRI administration on motor learning performance in health.

We do report evidence supportive of our second hypothesis, however, with significant decreases in functional responses in left premotor cortex during sequence-specific motor learning, relative to placebo (Figure 3). While both increases and decreases in functional brain responses underlie motor learning,^64^ this pattern is dependent on differential stages of learning and is defined by multiple parallel processes.^65–67^ Early fast learning is accompanied by rapid improvements in performance, followed by slow learning that characterizes a more consolidatory phase.^68^,^69^ Patterns of functional responses observed during this phase are also influenced by the type of task, with explicit learning of repetitive and unchanging sequences hypothesized to lead to faster automation of performance^66^,^70^,^71^ and a subsequent reduction in cognitive load needed for task completion. Given the predictable repetition of the learning sequence on our task and the timing of our assessments, it is possible that the observed escitalopram-induced decreases in the learning contrast reflect this automation of responses and subsequent consolidation of sequence learning.

Such a neural consolidation process in response to one week of escitalopram intake is consistent with a recent conceptual model of SSRI influences on post-stroke recovery.^72^ The authors propose that acute SSRI exposure changes the excitatory and inhibitory balance with increases in excitatory signaling, allowing for the remodeling of cortical pathways.^72^ Subsequent SSRI exposure leads to a reset in homeostasis with a heightening of GABAergic tone,^72^ allowing for remodeled pathways to become engrained as task performance continues. Further support for this interpretation stems from studies identifying an inverse relationship between cortical GABA concentrations and functional brain responses^73^,^74^ and SSRI administration has been shown to increase cortical GABA levels in rodents^75^ and healthy volunteers.^76^ Finally, the observation that the escitalopram-induced decrease in the learning contrast is negatively associated with escitalopram kinetics occurs in a timeframe consistent with that typically required for 5-HT_1A_ autoreceptor desensitization,^77^ which could also modulate effective enhancement of cortical GABAergic tone.^78^ In summary, it is possible that this escitalopram-induced decrease in premotor response in the learning contrast reflects more effective neural task processing, relative to placebo, in a region central to temporally and visually-oriented motor learning and planning.^79–82^ This interpretation is consistent with the hypothesis of an SSRI-induced window of experience-dependent plasticity as an attenuator of neural efficiency during performance.^25^,^26^

An alternative explanation of this finding is a habituation effect of neural responses during repetitive sequence-specific motor learning that may be emphasized by escitalopram administration. While we report a significant three-way interaction for brain, task, and group, this effect is limited to the comparison between a single dose and steady state and in the escitalopram group only, despite the observation that both groups successfully improve performance over time. It is possible that the neural responses during task performance in the placebo group reflect a simpler order effect, whereby neural responses adapt incrementally, rather than via an adaptive plasticity mechanism. Integration of more direct measures of cortical excitation and inhibition can allow for more fine-grained investigations into acute and subacute SSRI effects.

Nevertheless, there are some limitations to consider when interpreting these results. We acknowledge that the initial strong learning effect may have masked more subtle modulation of performance with escitalopram at a later training session. Though a known limitation of this task, we chose the SPFT for this well-established and reliable learning effect. While performance reaches a ceiling during the fourth and fifth sessions, as described previously,^40^ we still observe a considerable change in performance after the administration of the single dose and subsequent training sessions, thus maintaining the falsifiability of our primary hypothesis. Second, our results may not generalize to males or older adults as our sample consists only of females with standardized downregulation of ovarian hormones. This was a deliberate a-priori restriction to eliminate confounds such as sex differences^33^ and fluctuating endogenous sex hormones on environmental learning^83^ and escitalopram responsivity. While future studies in males, naturally cycling females, as well as healthy aged participants and stroke patients are needed, this choice of sample is also critical given that the majority of preclinical studies test only male samples,^84^ posing a critical obstacle to successful translation from healthy models to patients. Third, other studies have gradually increased escitalopram doses for pharmaco-fMRI protocols in healthy participants^85^ to minimize adverse effects. We chose a fixed dose of 20 mg to reliably block 80% of 5-HTT,^37^ an approach previously well tolerated.^28^ While four participants discontinued protocol because of adverse effects in the escitalopram group, this was also the case for two placebo participants, and there was no group difference in self-reported side effects at steady state. Finally, fMRI provides an indirect measure of neural activity, which is susceptible to non-neural changes such as vascular uncoupling. Given the functional specificity of the premotor cortex, it is unlikely that these findings are solely driven by changes in global blood flow. We cannot, however, identify underlying molecular mechanisms, which require quantitative measures such as MR-spectroscopy measures of GABA and glutamate or [^11^C]UCB-J positron emission tomography, a recently developed technique for in vivo imaging of synaptic plasticity.^86^,^87^

In conclusion, this is the first study to investigate the effect of steady state escitalopram administration on motor learning in an established sequential motor learning paradigm and the associated brain response in a sufficiently powered sample. In this pre-registered, randomized, controlled, interventional study, we do not find evidence in support of improved performance in response to one week of escitalopram intake during sequence-specific motor training. A major difference we observe between groups is a decrease in premotor cortical responses during sequence-specific learning performance comparing single dose and steady state. Considering previous findings on sequential motor learning and associated neural correlates in the motor network, less premotor response during similar performance may suggest more effective neural processing and greater consolidation of performance.^69^ By combining escitalopram administration and sequence-specific motor training for one week, we provide the first empirically tested framework for assessing SSRI effects on human adaptive motor plasticity in health. Our findings go beyond those of previous studies by employing a well-powered sample with combined subacute escitalopram administration and motor training, in a pre-registered design specifically tailored to empirically test the conceptual network hypothesis of SSRI action.^25^ These findings, therefore, address key methodological differences from previous studies in health and in stroke, while simultaneously providing an important step toward understanding the effects of SSRIs on human neural processing during sequence motor learning.

## Supplemental Material

sj-pdf-1-jcb-10.1177_0271678X20965161 - Supplemental material for Modulation of premotor cortex response to sequence motor learning during escitalopram intake Click here for additional data file. Supplemental material, sj-pdf-1-jcb-10.1177_0271678X20965161 for Modulation of premotor cortex response to sequence motor learning during escitalopram intake by Eóin N Molloy, Karsten Mueller, Nathalie Beinhölzl, Maria Blöchl, Fabian A Piecha, André Pampel, Christopher J Steele, Ulrike Scharrer, Gergana Zheleva, Ralf Regenthal, Bernhard Sehm, Vadim V Nikulin, Harald E Möller, Arno Villringer and Julia Sacher in Journal of Cerebral Blood Flow & Metabolism
