# Supplementary material for: Modulation of premotor cortex response to sequence motor learning during escitalopram intake
Source: J Cereb Blood Flow Metab. 2020 Nov 4;41(6):1449–62. doi: 10.1177/0271678X20965161 (PMC8138331; doi:10.1177/0271678X20965161)
Supplement: sj-pdf-1-jcb-10.1177_0271678X20965161 - Supplemental material for Modulation of premotor cortex response to sequence motor learning during escitalopram intake [file sj-pdf-1-jcb-10.1177_0271678X20965161.pdf]

Running Head: fMRI assessment of motor learning and escitalopram

Supplementary Materials

(i) Supplementary Methods:

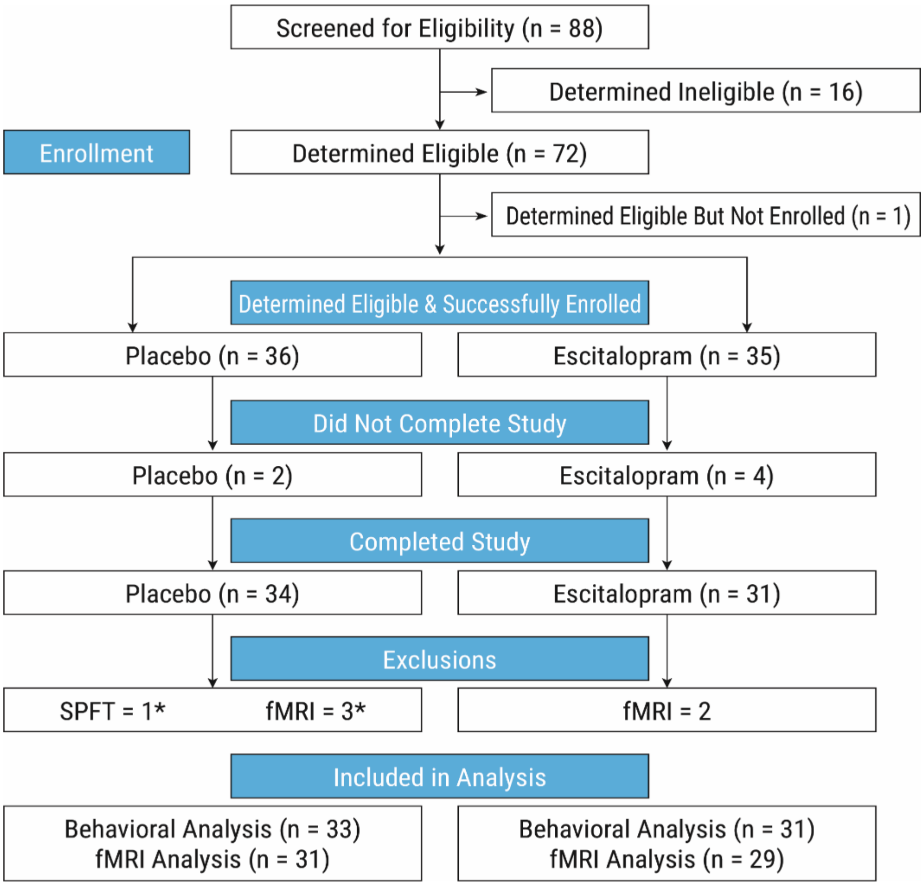

Supplementary Figure 1. Overview of study work-flow: This flow chart details the screening, inclusion, randomization, and exclusion procedure. Six participants did not complete the study protocol due to self-reported adverse effects (Placebo = 2/escitalopram = 4). Two participants who completed the protocol (Placebo = 1, SSRI = 1) were excluded due to excess head movement as calculated by framewise displacement (>3SD outside the mean), the presence of structural (placebo n=1) and functional (escitalopram n=1) image artifacts, and a pre-analytical error in plasma acquisition (n=1*). *Refers to the same participant excluded from both behavioral and fMRI analyses.

(ii) Supplementary Results

Supplementary Table 1.

Mean functional brain responses at baseline, single dose, and steady state for the escitalopram group (n=29). Results of a one-sample t-test of the escitalopram group, revealing mean functional response at each fMRI measurement. Results significant at p<0.001(unc) cluster forming threshold corrected with p<0.05 Family-wise error on the cluster level. p(FWE-corr) = p-value for coordinates within clusters formed at *p*<0.001(unc) that are significant with Family-wise error correction for multiple comparisons, Cluster Extent = number of voxels in cluster, MNI(x,y,z)=MNI peak coordinates. All results additionally Bonferroni corrected for multiple comparisons (α = 0.008).

Brain Region	*p(FWE-corr)*	*Cluster Size*	*t-value*	*z-value*	*MNI (x,y,z)*
Baseline					
Right Paracentral Lobule	<0.001	2774	6.42	4.99	9, -43, 74
			6.27	4.91	18, 2, 59
			6.18	4.87	-9, -49, 68
Left Cerebellum	<0.001	437	5.72	4.62	-42, -55, -34
			5.14	4.27	-36, -58, -52
			4.92	4.14	-33, -67, -49
Right Middle Temporal Gyrus	<0.001	478	5.68	4.6	48, -43, 5
			4.89	4.12	51, -46, 26
			4.81	4.07	51, -28, -4
Right Cerebellum	0.007	193	4.88	4.12	30, -40, -40
			4.53	3.89	30, -58, -34
			4.47	3.85	36, -52, -52
Single Dose					
Left Premotor Cortex	<0.001	14076	9.93	6.45	-24, 2, 62
			9.84	6.42	6, -52, 56
			9.62	6.34	-9, -49, 62
Right Cerebellum	<0.001	336	6.18	4.87	30, -67, -25
			6.05	4.8	36, -52, -49
			5.37	4.41	39, -58, -28
Steady State					
Right Postcentral Gyrus	<0.001	530	6.93	5.25	6, -49, 68
			6.05	4.8	-12, -49, 71
			4.54	3.9	-18, -64, 59
Left Cerebellum	0.001	310	5.78	4.65	-36, -49, -37
			5.38	4.42	-39, -58, -31
			4.86	4.11	-33, -70, -19

Supplementary Table 2. Mean functional whole brain responses at baseline, single dose, and steady state for the placebo group (n=31). Results of a one-sample *t*-test of the placebo group, revealing mean functional response at each fMRI measurement. Results significant at $p<0.001$(unc) cluster forming threshold corrected with $p<0.05$ Family-wise error on the cluster level. *p*(FWE-corr) = *p*-value for coordinates within clusters formed at $p<0.001$(unc) that are significant with Family-wise error correction for multiple comparisons, Cluster Extent = number of voxels in cluster, MNI(x,y,z)=MNI peak coordinates. All results additionally Bonferroni corrected for multiple comparisons ($\alpha = 0.008$).

Brain Region	*p(FWE-corr)*	*Cluster Size*	*t-value*	*z-value*	*MNI (x,y,z)*
Baseline					
Left Cerebellum	<0.001	8893	9.74	6.49	-36, -55, -49
			9.08	6.25	-24, -1, 65
			8.43	5.99	6, -52, 65
Right Cerebellum	0.001	198	7.05	5.37	30, -64, -52
			6.76	5.23	33, -52, -52
			6.57	5.13	27, -46, -46
Right Superior Temporal Gyrus	<0.001	1007	6.95	5.32	48, -28, 2
			6.86	5.28	51, -34, 8
			6.29	4.99	48, -37, 29
Single Dose					
Left Supp. Motor Area	<0.001	2646	7.82	5.73	-6, -1, 65
			6.63	5.16	24, 2, 65
			6.59	5.14	-24, 2, 68
Left Precuneus	<0.001	1010	7.52	5.59	-9, -58, 59
			6.96	5.33	6, -46, 74
			6.47	5.08	9, -52, 68
Left Cerebellum	<0.001	363	6.18	4.93	-39, -58, -31
			5.39	4.48	-30, -67, -25
			5.17	4.34	-36, -55, -43
Right Superior Temporal Gyrus	0.001	244	5.72	4.67	60, -40, 23
			5.05	4.26	48, -40, 2
			3.62	3.27	48 ,-31 ,-1
Steady State					
Left Postcentral Gyrus	<0.001	532	6.3	4.99	-9, -52, 65
			5.91	4.77	15, -61, 65
			5.26	4.39	-12, -61, 53
Left Premotor Cortex	0.001	251	6.21	4.94	-21, -1, 59
			5.6	4.6	-18, -1, 74
			4.51	3.91	-3, 5, 65
Right Middle Frontal Gyrus	0.007	168	5.75	4.68	30, 2, 65
			5.1	4.29	24, 2, 50
			4.51	3.91	18, 8, 62

Supplementary Table 3. Significant correlations of brain and behavior within the escitalopram group (n=29): Brain regions showing significant positive correlations between decreases in the learning contrast and improvements in lag learning-simple scores (LLSS) in the escitalopram group (n=29). Results significant at p<0.001(unc) cluster forming threshold corrected with p<0.05 Family-wise error on the cluster level. p(FWE-corr) = p-value for coordinates within clusters formed at *p*<0.001(unc) that are significant with Family-wise error correction for multiple comparisons, Cluster Extent = number of voxels in cluster, MNI(x,y,z)=MNI peak coordinates. Results additionally Bonferroni corrected for multiple comparisons (α = 0.01).

Brain Region	*p(FWE-corr)*	*Cluster Size*	*t-value*	*z-value*	*MNI (x,y,z)*
Left Premotor Cortex	<0.001	368	6.37	5.15	-18, -4, 56
			5.16	4.47	-18, 14, 53
			5.43	4.45	-9, 11, 68
Left Middle Temporal Gyrus	<0.001	373	5.70	4.61	-45, -64, -1
			5.13	4.27	-42, -79, 20
			4.69	4.21	-42, -55, 11
Left Middle Temporal Gyrus	<0.001	307	5.55	4.52	51, -55, -7
			4.86	4.11	39, -67, 26
			4.56	3.91	39, -55, 11
Left Postcentral Gyrus	<0.001	346	5.43	4.45	-12, -52, 59
			5.03	4.21	-6, -58, 65
			4.53	3.89	-15, -61, 53
Left Superior Frontal Gyrus	0.002	160	5.09	4.25	-12, -19, 41
			4.72	4.02	-6, -25, 53
			4.29	3.73	-12, -22, 59
Left Middle Frontal Gyrus	<0.001	338	4.86	4.11	-30, 44, 32
			4.62	3.95	-27, 53, 20
			4:60	3.94	-6, 41, 26
Left Thalamus	0.001	166	4.78	4.05	-18, -13, 14
			4.18	3.66	-27, -34, -4
			4.14	3.62	-18, -34, 19

Supplementary Table 4. Significant correlations of whole brain cortical motor response during sequence specific learning with plasma escitalopram levels in the escitalopram group (n=29): Brain regions showing significant negative correlations between decreases in the learning contrast and increases in escitalopram plasma levels. Results significant at p<0.001(unc) cluster forming threshold corrected with p<0.05 Family-wise error on the cluster level. *p*(FWE-corr) = *p*-value for coordinates within clusters formed at *p*<0.001(unc) that are significant with Family-wise error correction for multiple comparisons, MNI(x,y,z)=MNI peak coordinates. Results additionally Bonferroni corrected for multiple comparisons (α = 0.01).

Brain Region	*p(FWE-corr)*	*Cluster Size*	*t-value*	*z-value*	*MNI (x,y,z)*
Left Supramarginal Gyrus	0.002	164	5.87	4.70	-33, -40, 32
			5.15	4.29	-33, -43, 41
			4.26	3.71	-45, -40, 50
Right Posterior Somatosensory Cortex	<0.001	311	5.5	4.49	21, -55, 44
			5.31	4.38	33, -55, 26
			5.2	4.31	18, -61, 50
Left Premotor Cortex	<0.001	198	4.9	4.13	-9, 11, 68
			4.74	4.03	-21, -4, 68
			4.69	4.00	-18, -4, 56
